# Alum Pastilles

**Published:** 1860-04

**Authors:** 


### *Alum Pastilles.*

A Venetian physician, Dr. Argenti, proposes the use of alum pastilles, in place of alum solutions, prescribed as gargles in laryngo-pharyngeal anginas, in aphony and dysphony of singers, as well as for aphthous ulcerations of the mouth, whether these are simple, scorbutic, scrofulous, mercurial or typhoid. The formula is as follows:

℞.—Aluminis,
Gum Arab.,
Sacchari,
Water distilled several times with cherry laurel, ää, q. s.

To make pastilles weighing 40 centigrammes, (6 grains,) containing from 2 to 3 centigrammes of alum.

The mass, being well manipulated, and extended on a sheet of paper, cut up into pastilles and dried at a low heat, furnishes a preparation in which the astringent taste of the alum is mitigated by the other agents, and which can be preserved for some months. The pastilles are allowed simply to dissolve in the mouth, when the saliva bears the medicinal agent to the affected parts.—*Bulletin Gén. de Thérapeutique.*

